# Application of a high-throughput enantiomeric excess optical assay involving a dynamic covalent assembly: parallel asymmetric allylation and ee sensing of homoallylic alcohols†
†Electronic supplementary information (ESI) available. See DOI: 10.1039/c5sc02416a


**DOI:** 10.1039/c5sc02416a

**Published:** 2015-08-13

**Authors:** H. H. Jo, X. Gao, L. You, E. V. Anslyn, M. J. Krische

**Affiliations:** a Department of Chemistry , The University of Texas at Austin , Austin , Texas 78712 , USA; b Fujian Institute of Research on the Structure of Matter , Chinese Academy of Sciences , Fuzhou , 350002 , P. R. China . Email: lyou@fjirsm.ac.cn

## Abstract

Asymmetric Ir-catalyzed C–C coupling of primary alcohols with allyl-acetates, as described by Krische, to form chiral secondary homo-allylic alcohols were performed in parallel as a means to optimize the ee values thereof. Specifically, approximately 400 examples of this reaction were performed by varying the catalyst, added acids and bases, and starting reactants, to form 4-phenyl-1-butene-4-ol (**1**). The ee values for the transformations were determined in a high-throughput fashion using a 4-component assembly that creates a circular dichroism signal indicative of the extent of asymmetric induction. Further, a parallel and rapid quantitative TLC method measures the yield of each reaction, revealing which reactions give reliable ee values in the CD-based assay. Overall, the nearly 200 reactions whose ee values were determined could be quantitated in under two hours. Using a combination of the TLC method to measure yield with the CD-assay to measure ee values, several trends in reaction conditions were revealed. For example, it was found that the cyclometalated iridium catalyst modified by BINAP and *m*-nitro-*p*-cyano-benzoic acid delivered adduct **1** with the highest levels of enantiomeric enrichment (94%), whereas the corresponding SEGPHOS-modified catalyst gave a comparable yield but lower ee (91%). Most importantly, this study shows that supramolecular assemblies can report hundreds of ee values in a rapid and reliable fashion to analyze parallel synthesis routines.

## Abstract 2

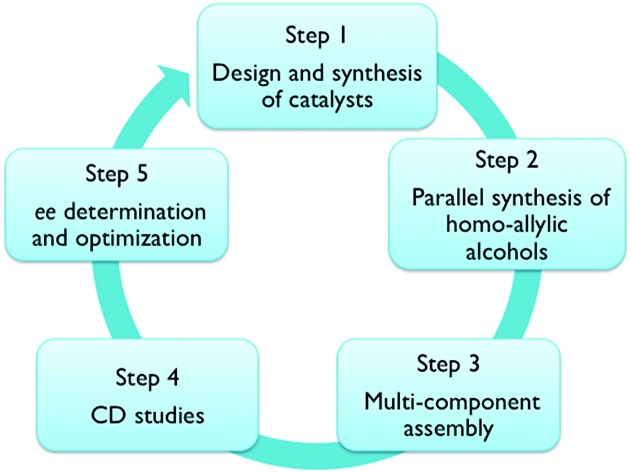
Parallel synthesis and high-throughput ee screening.

## Introduction

The asymmetric synthesis of enantiomerically enriched organic compounds is crucial in the pharmaceutical industry.^1^,^2^ For example, the asymmetric reduction of a ketone to an alcohol was used by Lilly to create Cymbalta, and an asymmetric enamine hydrogenation was used by Merck to generate Januvia.

Enantioselective metal catalysis ranks among the foremost methods used in the manufacture of chiral pharmaceutical ingredients.^3^–^6^ Asymmetric hydrogenation encompasses the majority of enantioselective metal catalyzed processes employed on scale.^5^,^6^ Indeed, the largest volume application of enantioselective metal catalysis is the asymmetric iridium catalyzed imine hydrogenation used to generate the agrochemical metolachlor, which delivers over 10 000 metric tons of chiral product annually.^7^,^8^ Inspired by the importance of asymmetric hydrogenation to the manufacture of chiral pharmaceutical and agrochemical products, the Krische group has developed a broad, new family of catalytic enantioselective C–C bond forming hydrogenations and transfer hydrogenations.^9^,^10^ Among these processes, the iridium catalyzed C–C coupling of primary alcohols and allyl acetate to furnish enantiomerically enriched homoallylic alcohols figures prominently,^11^–^15^ as it has been widely adopted in natural product synthesis.^16^–^24^

In any asymmetric reaction, the goal is to achieve as high enantiomeric excess (ee) as possible, which commonly involves testing numerous experimental conditions, reagents, and/or catalysts, to optimize the ee. This has led to the use of parallel synthesis and high-throughput screening (HTS) for reaction discovery.^25^ Due to utilization of micro-well plates, mini-block reactors and automated liquid handlers, these methods enable a series of reactions to be performed, varying any number of experimental parameters and reagents, in a short amount of time. Conventionally for ee determination, chiral HPLC and GC are used. However, they are not suitable for HTS of ee when hundreds to even thousands of reactions are to be analyzed. For this reason, optical assays for ee determination using colorimetric,^26^–^28^ fluorescence,^29^,^30^ or circular dichroism (CD) spectroscopy,^25^,^31^,^32^ are being created for the interrogation of chiral organic compounds.

The Anslyn group has reported several CD methods using dynamic assemblies for various chiral functional groups: amines, alcohols, carboxylic acids, and aldehydes/ketones.^33^–^36^ Each assay was validated with a limited number of commercially available chiral examples. The assays are not as accurate as conventional chromatographic techniques, having errors ranging between 3 and 7%. However, such errors are deemed sufficient for a pre-screening of asymmetric reactions to discover trends or uncover hits. These errors are acceptable primarily due to the ease of use of the assays and their speed. For example, in each assay the chiral analyte is simply mixed with the assembly components, the solutions are allowed to reach equilibrium, and a robot transfers the samples from a 96-well plate to a CD spectrometer for the determination of 96 ee values in under two hours.^37^ Alternatively, when using a laser table with four photo-elastic modulators, such a plate can be read in less than five minutes.^38^ While the Anslyn amine and alcohol assays have been used by Zhang^39^ and Miller,^40^ respectively, to measure a few dozen ee values each,^41^ we have yet to truly put the assays to the test measuring hundreds of values.

In this article, we describe our first successful combination of parallel reactions with rapid CD analysis. We describe how the Krische methodology for generating homo-allylic alcohols can be combined with our HTS ee assay for chiral alcohols.^34^ Furthermore, we also have developed a quantitative Thin Layer Chromatography (TLC) analysis to avoid analyzing reactions with low yields, regardless of their ee values. In total, approximately 400 reactions were analyzed, with nearly 200 ee values being determined. Once the assays were considered functional, the total analysis time for combined TLC and ee screening could be accomplished in under a day. The ee screening alone was easily performed in under four hours. The study validates that the assays are functional in a real-life setting.

## Results and discussion

### Iridium catalyzed carbonyl allylation reactions in 96-well mini-block

The Krische group has reported the iridium catalyzed enantioselective alcohol-mediated carbonyl allylation shown in eqn (1). Initially, *in situ* assembly of the iridium catalysts were performed due to convenience.^42^ In their later work, isolable single iridium catalysts, referred to as “preformed catalysts”, were explored.^43^,^44^ Using the preformed catalysts was advantageous in terms of yield and could be used under milder conditions. Thus, in our studies we adopted the use of preformed catalysts.1
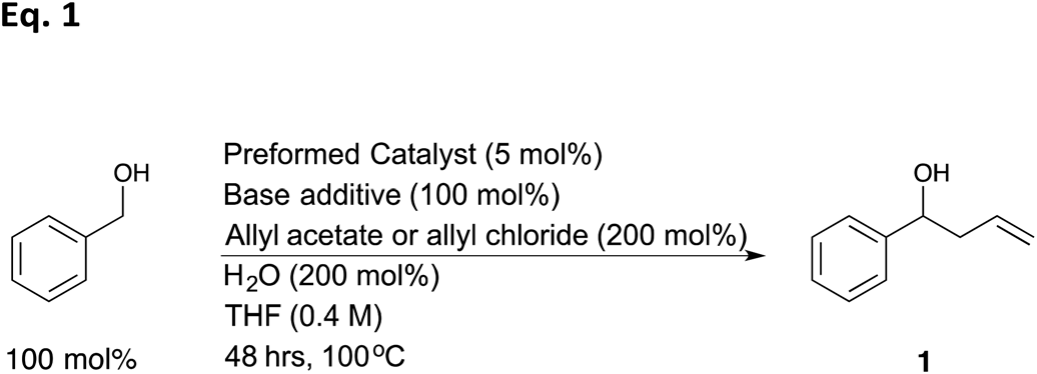



In the present study, 18 preformed catalysts were generated by varying the acid and the chiral phosphine ligands as shown in eqn (2) (Table 1, and enantiomers of each entry). Cesium carbonate as a base and allyl acetate were used for all preformed catalysts. Based upon previous results from the Krische group, the chiral phosphine SEGPHOS generally gives the highest ee values in the carbonyl allylation. But, due to the fact that BINAP is much less expensive, a major focus of this project was to screen reactions in an attempt to reveal conditions where BINAP would rival SEGPHOS. Previous studies also give the highest production yield of catalyst when a meta-substituted carboxylate was used. Thereby, we set out to explore *m*-nitrobenzoic acids with various electron-withdrawing and electron-donating groups on the *para* position.

**Table 1:** Various conditions creating 18 combinations of catalysts, as shown in eqn (2)

Entry	Ligand^*a*^	Acid	Preformed catalyst
1	R-BINAP	A1	C1
2	R-BINAP	A3	C2
3	R-BINAP	A5	C3
4	R-SEGPHOS	A1	C4
5	R-SEGPHOS	A3	C5
6	R-SYNPHOS	A1	C6
7	R-SYNPHOS	A3	C7
8	R-Cl, MeO-BIPHEP	A1	C8
9	R-Cl, MeO-BIPHEP	A3	C9

^*a*^ *S*-enantiomers of the ligands were also used to generate pre-catalysts.

Using the 18 catalysts described, as well as a series of other experimental conditions (described below), we performed nearly 400 carbonyl allylations that create 4-phenyl-1-butene-4-ol (eqn (1)). For this parallel synthesis, a series of preformed catalysts, bases and solvents were screened in 96-well reaction-blocks, sometimes loaded with only 48 reactions or other times fully loaded, each well being a different reaction condition associated with its position on the plate. Often, when double-checking results, the plates were loaded with only 20 reactions. The reactions were stirred for two days, heated with two different procedures. In some cases the reaction-block was sealed, buried in a sand bath, and heated at 100 °C with a flea stir-bar in each well. Alternatively, the reaction-blocks could be placed directly on a heat plate with a temperature controller embedded in the reaction block. Both procedures gave consistent results. After two days the blocks were opened, and purification was performed using a parallel procedure involving various 96-well filter and collection plates. First, a small spatula of silica was added to each well (approximately 200 mg), followed by simultaneous removal of the solvent from all wells using a Genevac (dry loading method). Second, each well of a 96-well filter plate was loaded with approximately 1 cm of silica. Each crude reaction mixture was loaded into this filter plate using the same positions as in the reaction plate. Approximately 2 ml of 2% EtOAc in hexanes, followed by about 2 ml of 5% EtOAc, was flushed through each well in the filter-plate and collected in deep-well plates. The first wash removes the excess benzyl alcohol, whereas the second wash elutes the product. The benzyl alcohol must be removed because it can compete with the chiral secondary alcohol in the assay that reports the ee values (*vida infra*). The allyl moiety, either acetate or chloride, can co-elute with the product alcohol because we found it has no influence on the ee determination. The solvent of the product-containing fractions from the second wash was removed again using a Genevac. At the end, homo-allylic alcohol **1** was the only compound that remained in the 96-well plate that responds in our ee assay.2
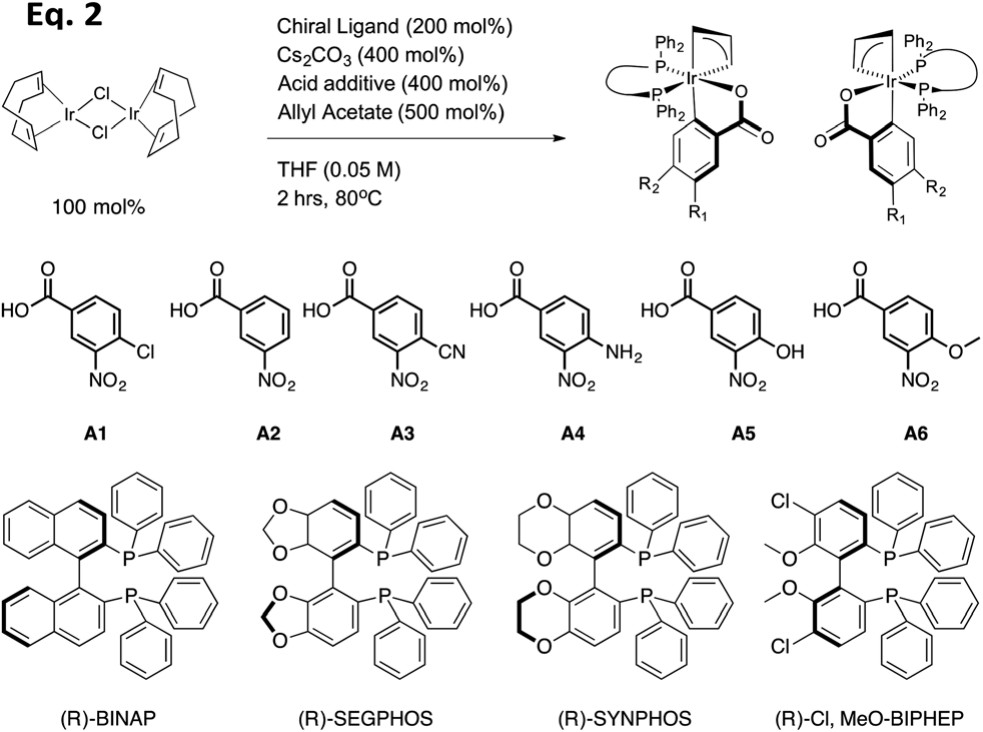



### ee Sensing of homo-allylic alcohols using a dynamic multi-component assembly

**Scheme 1:**
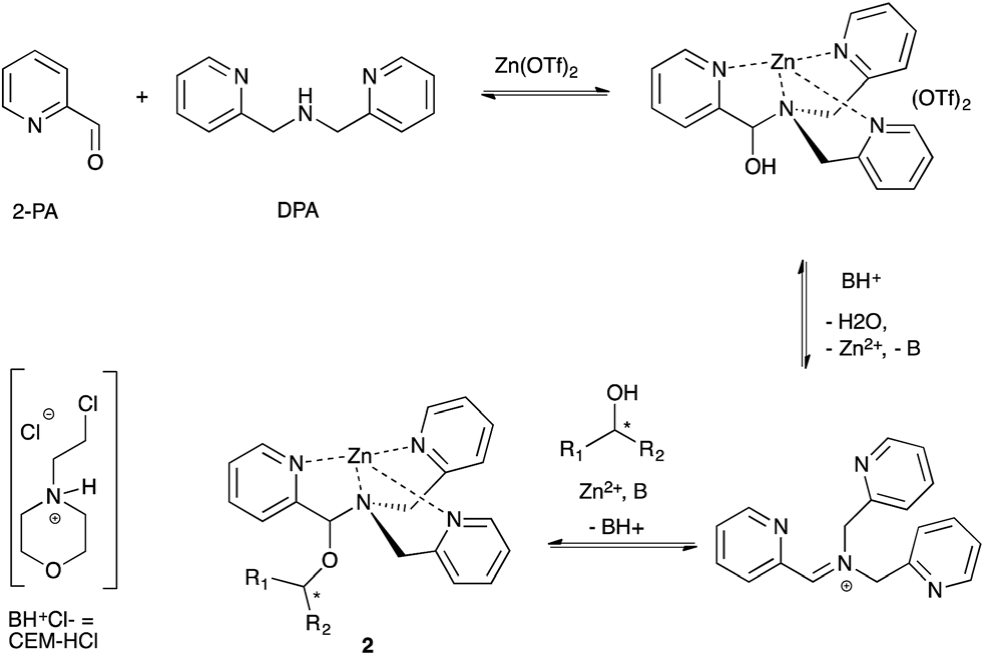
Four-component assembly that reports the ee values of chiral secondary alcohols *via* examination of the resulting CD spectra.

Our group previously reported an assay to measure the ee values of chiral secondary alcohols using a four-component assembly. The assembly arises from a combination of 2-picolinaldehyde (2-PA), di-(2-pyridylmethyl)amine (DPA), an alcohol and a zinc salt, creating a tris-pyridine inorganic coordination complex **2** (Scheme 1).^34^,^45^ In the tris-pyridine complex, a hemi-aminal ether functional group exists, which induces a helical twist in the pyridine ligands, thereby inducing a large cotton effect in the CD spectrum. Most chiral homo-allylic alcohols exhibit very weak or no signals in the CD spectrum. Thus, because our dynamic multi-component assembly creates large CD signals at wavelengths where the products do not absorb, we are in essence amplifying the signal. Although this system has been successfully used to quantify the ee values of a variety of commercially available chiral secondary alcohols, the assay had yet to be used in a true high-throughput manner.

**Fig. 1:**
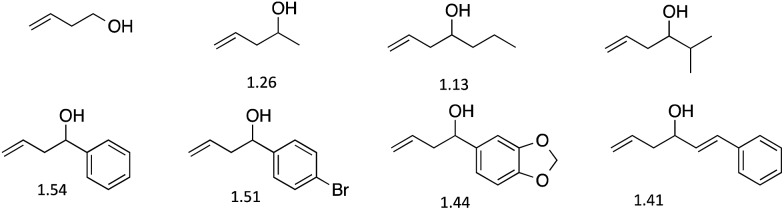
Various homoallylic alcohols studied and their d.r. values.

We first analyzed a series of homo-allylic alcohols to verify that our assay would function with such structures (Fig. 1). The four-component assembly using each alcohol was studied by ^1^H NMR and ESI-mass spectroscopy. The presence of diastereomers of **2** formed from the homo-allylic alcohols was confirmed by ^1^H NMR spectroscopy. Diastereomeric ratios (d.r.) were measured by integrating the hydrogen resonances of the chiral alcohols within the assembly (Fig. 1). In the past, we have been able to create successful ee assays for alcohols with d.r. values as small as 1.2, and therefore every analyte examined would be successful in our assay.^46^

**Fig. 2:**
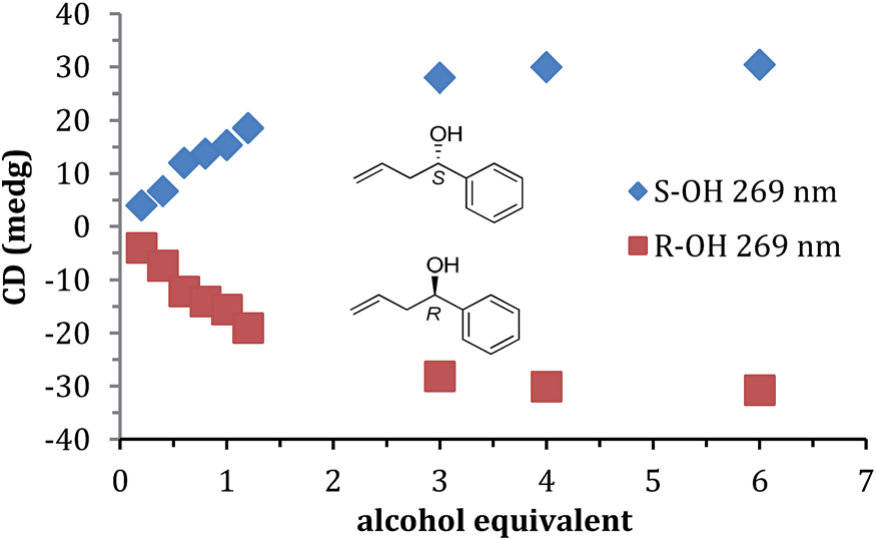
Titration of alcohol **1** into 2-PA, DPA, and Zn(ii) triflate (all at 269 nm) in acetonitrile.

A titration was performed with **1** to find the minimum concentration required to reach signal saturation (Fig. 2). Three equivalents were sufficient, thus removing concentration dependence at this or high equivalents. Therefore, to create calibration curves that relate CD ellipticities to ee, 3 equivalents of **1** relative to the assembly was used. It is important to note that during the parallel reaction screening, if the yield of the alcohol was such that it had been lower than 3 equivalents, then the ee values determined would be incorrect. Therefore, in many cases (as described below), we determined the reaction yield prior to measuring the ee values simply to verify that we could trust the ee values from our assay.

**Fig. 3:**
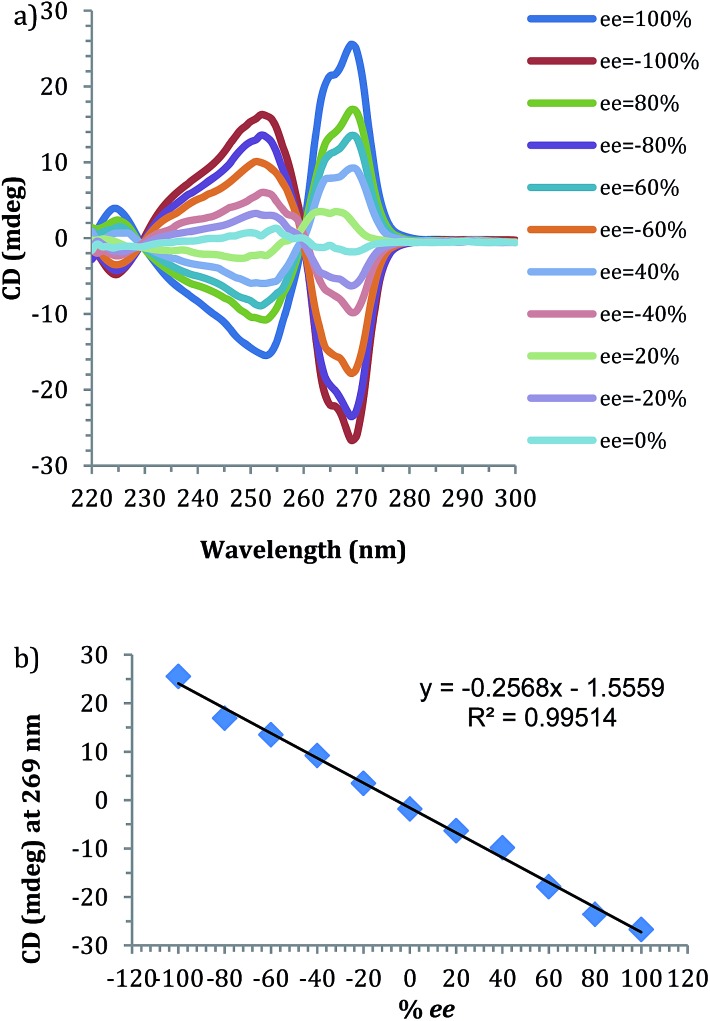
(a) CD spectra of 4-phenyl-1-butene-4-ol derived assembly with different ee of alcohol (0.175 mM 2-PA, 0.525 mM alcohol). (b) Linear ee calibration curve lines at 269 nm.

Prior to the anticipated parallel screening, a calibration curve for 4-phenyl-1-butene-4-ol (**1**) was generated using the CD spectra of the multi-component assembly reaction (Fig. 3a). The calibration curve (Fig. 3b) showed a correlation coefficient of 0.99.

The approximately 400 samples of **1**, created using the parallel synthesis routine described above, were analyzed in numerous 96-well plates employing the multi-component assembly of **2PA**, Zn(OTf)_2_, **DPA** and CEM-HCl (Scheme 1). The analyses were performed *in situ*, in parallel, in acetonitrile, and in the presence of molecular sieves. The well plates were sealed with 96-well strip plates, and the mixtures were stirred at room temperature for ∼18 h, allowing the assembly equilibria to be achieved. A CD spectrum of each well was taken and the ee was determined using the calibration curve generated for **1**. Using a robot, the plate could be read in 2 hours, but using a newly developed CD laser system, each plate can be read in 5 minutes.^38^

### TLC imaging to pre-screen allylation reactions

As described above, a caveat for this parallel synthesis and the HTS ee assay is that a minimum of 3 equiv. of alcohol is needed to ensure saturation of the CD signal. If the reaction gives a low yield, then even if the reaction gives high enantioselectivity, the CD reported ee values will not be accurate. To circumvent this issue, we devised a rapid and parallel TLC method to roughly quantify the yield of each reaction.^47^–^50^ The experiment was set up such that if 100% yield of **1** (eqn (1)) were achieved, that would represent 6 equivalents to be used in the assembly that measures ee values, hence far beyond the 3–4 equivalents needed to saturate the CD signal. Therefore, even around a 60–65% yield would still give saturation of the CD signal and an accurate ee value. Hence, the assay created to measure yields needed to have a threshold of detection of 60% with a ±10% error. The yield can be calculated if we quantify the ratio between the starting precursor, benzyl alcohol, and the product alcohol, 4-phenyl-1-butene-4-ol. If the yield was below 60%, further detection of the ee was not performed.

Benzyl alcohol and 4-phenyl-1-butene-4-ol are both luminant under a UV lamp when spotted on a silica gel plate. Microcaps were used to spot the reactions on the TLC plates. A box was constructed that held an iPhone with a hole for the camera, and a UV lamp was set to uniformly illuminate the TLC plate from one side of the box. The camera was placed the same distance from TLC plate as the lamp because we found this was best at making the image not too bright or too dim.

**Fig. 4:**
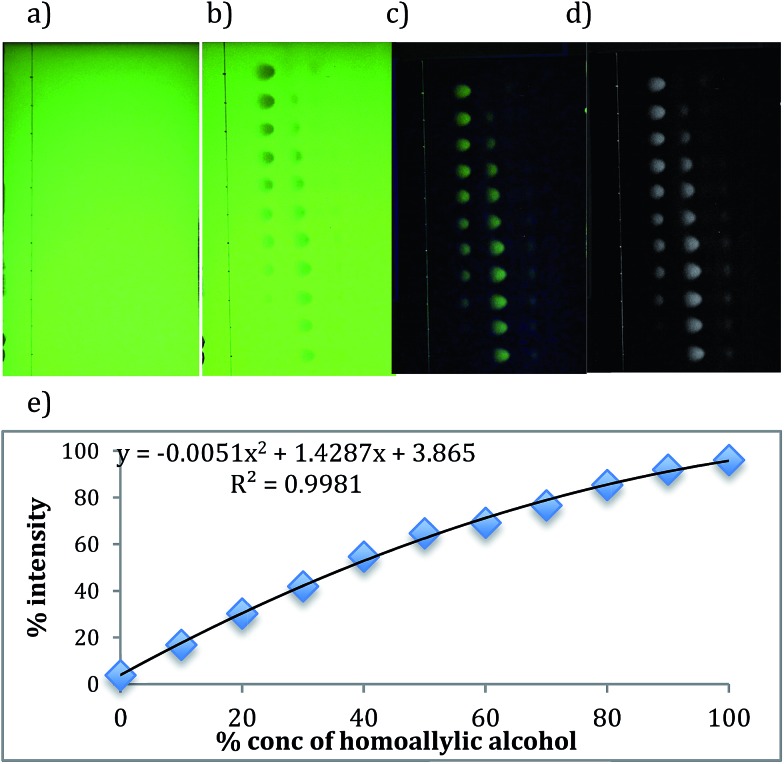
(a) Raw image of blank TLC plate under UV lamp (b) raw image of TLC plate spotted with different ratios of starting and product alcohol under UV lamp (c) image of TLC plate when background is subtracted (d) background subtracted image of TLC plate in grey scale (e) calibration generated from % concentration of product alcohol to starting alcohol *vs.* % product alcohol intensity counts for each development.

To generate calibration curves to quantify the yield of the reactions, images of empty TLC plates were taken (Fig. 4a). These are needed for subtracting the background when analyzing with computer software. Next, 11 samples that contain different ratios of starting and product alcohols, all at the same total concentration identical to that used in the parallel reactions, were spotted on the plates. The plates were allowed to dry and eluted in 20% ethyl acetate in hexane. The developed TLC plate was placed in the same location where the empty TLC plate was placed before, and the image was taken (Fig. 4b).

The software Igor pro was used to analyze the images. By subtracting the background image, only benzyl alcohol and 4-phenyl-1-butene-4-ol spots appear (Fig. 4c). After converting color to black and white (Fig. 4d), the program counts the pixels of each spot and takes ratios of counted pixels, thereby generating a calibration curve (Fig. 3, Table 3). The graph is slightly curved, due to the fact that at the low end the program picks up some signal for **1**, even though there should be none (note that the graph does not start at zero on the *y*-axis). At the high end, the program detects some signal for benzyl alcohol although there is none (note that the values are less than 100% on the *y*-axis). Because the same issues will exist when analyzing hundreds of parallel reactions, we used a curve to fit the calibration data. This screening step, using simple TLC techniques, proved extremely useful. It was used either to eliminate some reactions before ee determination, or for checking the yields of reactions after ee determination when the CD signals were very low implying either a low yield or poor enantioselectivity.

### Determination of ee of homo-allylic alcohol

Combinations of 9 different pre-catalysts, their enantiomers, 4 different bases, 3 different solvents, and 2 different allyl moieties generated more than 400 reactions that were ran in parallel. Their yield and ee was checked rapidly using our CD method (see ESI†). For randomly selected reactions, HPLC determined ee and CD determined ee was compared and gave average errors of ±7% for examples that give a yield of **1** above 60% (Table 2). For the pre-catalyst, using BINAP as a chiral ligand along with *m*-NO_2_–*p*-CN–BzOH showed the highest ee of the product alcohol **1** (94%). Using SEGPHOS as a chiral ligand also gave comparable yield as well as an ee of 91%. The sample using BINAP with a 94% CD-determined ee value was checked *via* HPLC, revealing an ee of 98%. This is above the 94% ee found for catalysts incorporating SEGPHOS determined using HPLC (Table 2, and ESI†). Thus, our screening revealed conditions that revealed that the less expensive ligand could rival SEGPHOS. Among the acids (A1–A6) that were used to generate preformed catalysts, having more electron-withdrawing groups *para* to the benzoic acid gave the best yield. Among inorganic bases, changing bases in the reaction resulted in differences in yield but did not significantly change the ee. But there was a decrease in ee with the chiral base. Change in allyl moiety did not influence the yield or ee of **1**. A sample with a reasonably high yield that was confirmed by TLC, the ee determined by CD was in a small error range with the ee determined by HPLC.

**Table 2:** Various conditions creating 18 combinations of catalysts, as shown in eqn (2)

Pre-catalyst	Allyl moiety	Base	Solvent	CD ee	HPLC ee	TLC yield (%)	Isolated yield (%)
C1^*a*^	1a^*b*^ (allyl acetate)	Cs_2_CO_3_	THF	90	92	80	85
			MeCN	8	54	25	18
			Dioxane	88	90	68	52
		K_2_CO_3_	THF	45	77	48	58
			MeCN	11	47	15	12
			Dioxane	65	68	32	25
		Strycine	THF	18	51	22	43
		K_2_CO_3_	THF	45	77	48	58
C2	1a	Cs_2_CO_3_	THF	94	98	85	84
	1b			90	94	85	—
C5	1a	Cs_2_CO_3_	THF	89	94	80	78
	1b			90	94	75	80

^*a*^C2–C9 and its enantiomers (see Table 1) were also performed with above conditions (see ESI for more details).

^*b*^1b (allyl chloride) was also performed with above conditions with C1–C9 and its enantiomeric catalysts.

**Table 3:** Isolated yield and TLC method based calculated yield varying base and acid additives

Entry	Base	Acid	% Yield	% Yield (calcd)
1	Cs_2_CO_3_	A1	85	80
2	Cs_2_CO_3_	BzOH	44	48
3	Na_2_CO_3_	A1	16	23
4	K_2_CO_3_	A1	24	27
5	Cy_2_NMe	A1	—	—
6	Strychnine	A1	22	43
7	Brucine	A1	—	—

It is important to note that when a low yield of alcohol was observed *via* the TLC assay, the CD determined and HPLC ee values showed quite a discrepancy (Table 2). Thus, one limitation of our rapid CD-based assays is that highly asymmetric transformations that are low yielding will be missed due to the fact that they would be inaccurately reported. Yet, we feel that the advances reported here using a simple and quantitative TLC-approach will help to rule out miss-interpretations because the low-yielding reactions can be identified before CD-analysis.

## Conclusions

We successfully demonstrated parallel enantioselective carbonyl allylation reactions using multi-well mini-blocks followed by ee determination using a dynamic multi-component CD assay. By performing the synthesis of the analytes as well as sensing assemblies in multi-well plates, we have achieved true HTS. The overall result shows that when the reaction gives a reasonable yield (>60%), the CD based ee assay gives acceptable errors. However, when the yield of **1** is low, because saturation is not reached in the multi-component assembly, the ee values are inaccurate. Our screen revealed conditions for the specific analyte **1** that allowed the less expensive BINAP ligand to rival SEGPHOS, a ligand that is found to generally give high ee for other similar carbonyl allylations.^42^,^43^ Similarly, each of our group's HTS assays should allow chemists to analyze hundreds of reactions that create the same chiral structure using the same calibration curve for CD *versus* ee values.

## Supplementary Material

Supplementary information
