# Supplementary material for: Application of a high-throughput enantiomeric excess optical assay involving a dynamic covalent assembly: parallel asymmetric allylation and ee sensing of homoallylic alcohols
Source: Chem Sci. 2015 Aug 13;6(12):6747–53. doi: 10.1039/c5sc02416a (PMC4800411; doi:10.1039/c5sc02416a)
Supplement: Supplementary file 1 [file SC-006-C5SC02416A-s001.pdf]

Electronic Supplementary Material (ESI) for Chemical Science.
This journal is © The Royal Society of Chemistry 2015

Supporting Information

Application of a High-Throughput Enantiomeric Excess Optical Assay Involving a Dynamic Covalent Assembly: Parallel Enantiomeric Allylation and *Ee* Sensing of Homoallylic Alcohols †

Hyun Hwa Jo, Xin Gao, Lei You, Eric V. Anslyn* and Michael J. Krische*

<u>Content</u>

General Information S1

Experimental Procedures

Preparation of pre-catalysts S2

General Procedures for Synthesis of 1-Phenylbut-3-en-1-ol (**1**) S4

General Procedures for Multi-component Assembly

General Procedures for TLC Analysis S5

Tables S6

General Information:

NMR spectra were recorded on Agilent MR 400 at The University of Texas at Austin NMR facility. ESI-mass spectra were obtained on Agilent 6100 at The University of Texas at Austin mass spectrometry facility. Circular dichroism (CD) spectra were recorded on a Jasco J-815 spectropolarimeter at The University of Texas facility. High performance liquid chromatography (HPLC) analysis was performed with OD-H column from Chiralcel.

Experimental Procedures:

Preparation of pre-catalysts

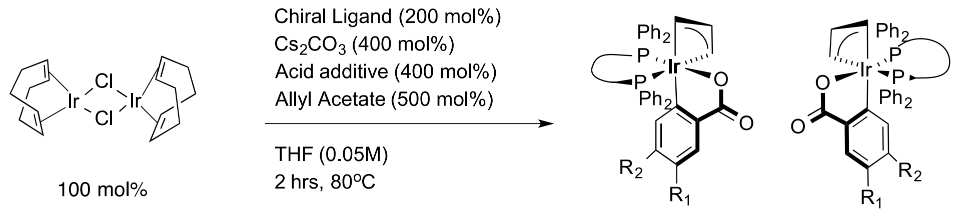

Sheme S2. Synthesis of pre-catalysts with various phospho ligands

A pressure tube equipped with a magnetic stirbar was charged with $[Ir(cod)Cl]_2$ (161.9 mg, 0.24 mmol, 100 mol%), phospho ligand (0.48 mmol, 200mol%), Cs_2CO_3 (312.9 mg, 0.96 mmol, 400 mol%) and 4-Cl-3-NO_2BzOH (192.5 mg, 0.96 mmol, 400 mol%). The tube was purged with argon and an outlet for 5 minutes, followed by the addition of allyl acetate (142μL, 1.2 mmol, 500 mol%) in THF (5.2 mL, 0.05 M). The mixture was stirred for 30 min at room temperature under argon. The reaction mixture was then stirred for 90 min at 80 °C. Upon cooling to ambient temperature, the reaction mixture was diluted and rinsed with CH_2Cl_2 and concentrated in vacuo at ambient temperature. The compound was purified by flash chromatography (SiO_2, 20% Et_2OAc/CH_2Cl_2) and concentrated in vacuo at ambient temperature. The light brown gum was dissolved in small amount of THF and precipitated upon rapid addition of HPLC grade hexanes. Gravity filtration, followed by removal of trace solvents with flushing N_2 gives a yellow powder.

Table S3. List of pre-catalyst generated and the yield

Pre-catalyst	Phospho ligand	Acid	Yield (%)
(R)-C1	(R)-BINAP	4-Cl-3-NO_2BzOH	80
(R)-C2	(R)-BINAP	4-CN-3-NO_2BzOH	78
(R)-C3	(R)-BINAP	4-OH-3-NO_2BzOH	66
(R)-C4	(R)-SEGPHOS	4-Cl-3-NO_2BzOH	76
(R)-C5	(R)-SEGPHOS	4-CN-3-NO_2BzOH	83
(R)-C6	(R)-SYNPHOS	4-Cl-3-NO_2BzOH	76
(R)-C7	(R)-SYNPHOS	4-CN-3-NO_2BzOH	79
(R)-C8	(R)-Cl, MeO-BIPHEP	4-Cl-3-NO_2BzOH	79
(R)-C9	(R)-Cl, MeO-BIPHEP	4-CN-3-NO_2BzOH	81
(S)-C1	(S)-BINAP	4-Cl-3-NO_2BzOH	81
(S)-C2	(S)-BINAP	4-CN-3-NO_2BzOH	80
(S)-C3	(S)-BINAP	4-OH-3-NO_2BzOH	65
(S)-C4	(S)-SEGPHOS	4-Cl-3-NO_2BzOH	76
(S)-C5	(S)-SEGPHOS	4-CN-3-NO_2BzOH	80
(S)-C6	(S)-SYNPHOS	4-Cl-3-NO_2BzOH	78
(S)-C7	(S)-SYNPHOS	4-CN-3-NO_2BzOH	78
(S)-C8	(S)-Cl, MeO-BIPHEP	4-Cl-3-NO_2BzOH	80
(S)-C9	(S)-Cl, MeO-BIPHEP	4-CN-3-NO_2BzOH	82

General Procedures for Synthesis of 1-Phenylbut-3-en-1-ol (**1**)

To a vial in the mini-block, pre-catalyst (8.75 μmol, 5 mol%) and stock solutions of benzyl alcohol (17.5 μmol, 100 mol%), base (10.5 μmol, 6 mol%) in THF (0.4 M) and H_2O (0.35 mmol, 200 mol%) was placed. The stock solution of allyl acetate (0.35 mmol, 200 mol%) in THF was added in the mixture and was allowed to stir at 100 ^oC for 48 hr, at which point the reaction mixture was evaporated onto silica gel using Genevac. Purification of the product by mini-plug silica column chromatography on 96-well filter plate (SiO_2: ethyl acetate:hexanes, 1:50 to 1:20) provided **1** as a colorless oil. Spectroscopic properties matched those in the literature[11,12].

HPLC: (Chiralcel OD-H column, hexanes:i-PrOH = 95:5, 0.5 mL/min, 254 nm), (R)-1-Phenylbut-3-en-1-ol: 17.2 min, (S)-1-Phenylbut-3-en-1-ol: 15.2 min.

General Procedures for Multi-component Assembly

All assembly reactions were performed using stock solution of each component *in situ* in acetonitrile without isolation and purification. Pyridine-2-carboxyaldehyde (**2-PA**, 35 mM, 0.0175 mmol, 1 equiv.), zinc triflate ($Zn(OTf)_2$, 35 mM, 0.0175 mmol, 1 equiv.), di-(2-picolyl)amine (**DPA**, 42mM, 0.021 mmol, 1.2 equiv.), 1-phenylbut-3-en-1-ol (**1**, equivalents varies within allylation reactions performed.), and 4-(2-chloroethyl)morpholine hydrochloride (CEM-HCl, 35 mM, 0.0175 mmol, 1 equiv.) were stirred together in acetonitrile in the presence of 3Å activated molecular sieves. The mixture was stirred at room temperature.

General Procedures for TLC Analysis

Each reaction was spotted onto TLC silica plate and developed with 10% ethylacetate in hexane. The TLC plate was placed inside the box that has UV lamp attached and the photo was taken using iphone. It is important in each time, the TLC plate to be placed at the same position and that a picture of blank TLC plate need to taken before the development. The pictures were converted to grey-scale using any kind of photo management software, then using a software Igor pro, the intensity for each spot was counted and the ratio between the starting alcohol and the product alcohol was calculated as percentile.

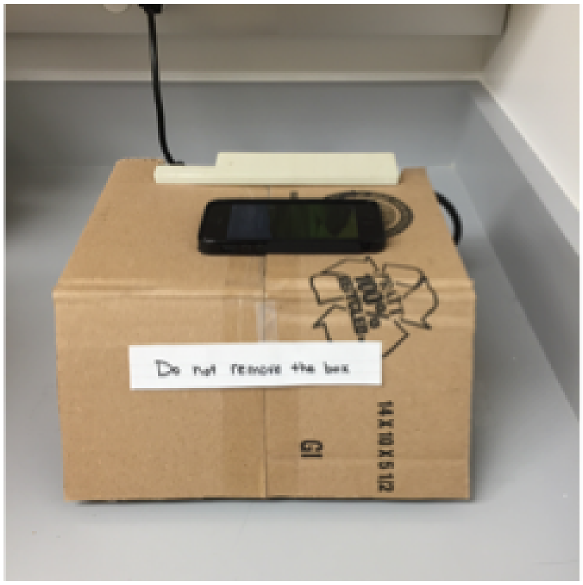

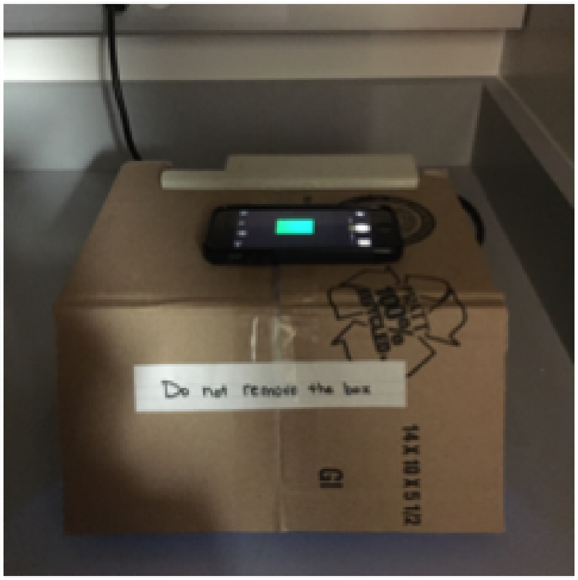

Tables of *ee* and % yield of 1 for each reaction

Allylation was performed for each pre-catalyst varying allyl moiety, base and solvent to give product alcohol **1** followed by multi-component assembly. The *ee* obtained from a CD based calibration curve and yield estimated using the TLC method for each condition are shown below. Some of the reactions were randomly selected to compare with HPLC *ee* and isolated yield. When using enantiomers of the pre-catalysts, essentially the opposite *ee* were obtained with slight fluctuations in yield.

Pre-catalyst	Allyl Moiety	Base	Solvent	CD *ee*	HPLC *ee*	TLC yield (%)	Isolated yield (%)
(R)-C1	1a	Cs_2CO_3	THF	90	92	80	85
			MeCN	8	54	25	18
			Dioxane	88	90	68	52
		K_2CO_3	THF	45	77	48	58
			MeCN	11	47	15	12
			Dioxane	65	68	32	25
		K_3PO_4	THF	85	90	80	80
			MeCN	12	48	25	23
			Dioxane	88	89	83	78
		strycine	THF	18	51	22	43
			MeCN	7	42	16	12
			Dioxane	12	38	33	48
(R)-C2	1a	Cs_2CO_3	THF	94	98	85	84
	1b			90	94	85	-
	1a	K_3PO_4		90	84	80	-
(R)-C4	1a	Cs_2CO_3		88	93	75	80
	1b			87	93	78	76
(R)-C5	1a	Cs_2CO_3		89	94	80	78
	1b			90	94	75	80
	1a	K_3PO_4		91	96	70	-
(R)-C6	1a	Cs_2CO_3		86	84	82	77
	1b			85	84	77	-
(R)-C8	1a	Cs_2CO_3		84	86	80	75
	1b			84	85	75	-

Pre-catalyst	Allyl Moiety	Base	Solvent	CD *ee*	TLC yield (%)
(R)-C1	1a	Cs_2CO_3	THF	90	80
			MeCN	8	25
			Dioxane	88	68
		K_2CO_3	THF	45	48
			MeCN	11	15
			Dioxane	65	32

		K_3PO_4	THF	85	80
			MeCN	12	25
			Dioxane	88	83
		strycine	THF	18	22
			MeCN	7	16
			Dioxane	12	33
	1b	Cs_2CO_3	THF	93	68
			MeCN	14	20
			Dioxane	90	65
		K_2CO_3	THF	58	52
			MeCN	18	24
			Dioxane	35	28
		K_3PO_4	THF	92	80
			MeCN	20	28
			Dioxane	88	64
		strycine	THF	19	24
			MeCN	11	25
			Dioxane	16	24

Pre-catalyst	Allyl Moiety	Base	Solvent	CD *ee*	TLC yield (%)
(R)-C2	1a	Cs_2CO_3	THF	94	85
			MeCN	12	20
			Dioxane	86	65
		K_2CO_3	THF	52	61
			MeCN	14	10
			Dioxane	68	52
		K_3PO_4	THF	90	80
			MeCN	21	28
			Dioxane	92	77
		strycine	THF	20	25
			MeCN	12	14
			Dioxane	12	18
	1b	Cs_2CO_3	THF	90	85
			MeCN	7	12
			Dioxane	90	72
		K_2CO_3	THF	33	43
			MeCN	7	12
			Dioxane	68	48
		K_3PO_4	THF	88	87
			MeCN	15	22
			Dioxane	88	75
		strycine	THF	18	14
			MeCN	10	12
			Dioxane	21	34

Pre-catalyst	Allyl Moiety	Base	Solvent	CD *ee*	TLC yield (%)
(R)-C3	1a	Cs_2CO_3	THF	85	74
			MeCN	15	22
			Dioxane	78	70
		K_2CO_3	THF	65	51
			MeCN	14	22
			Dioxane	64	52
		K_3PO_4	THF	70	80
			MeCN	14	28
			Dioxane	67	58
		strycine	THF	15	22
			MeCN	8	12
			Dioxane	12	12
	1b	Cs_2CO_3	THF	87	80
			MeCN	12	19
			Dioxane	87	70
		K_2CO_3	THF	49	44
			MeCN	10	18
			Dioxane	69	52
		K_3PO_4	THF	80	70
			MeCN	14	22
			Dioxane	78	68
		strycine	THF	11	28
			MeCN	6	10
			Dioxane	10	23

Pre-catalyst	Allyl Moiety	Base	Solvent	CD *ee*	TLC yield (%)
(R)-C4	1a	Cs_2CO_3	THF	88	75
			MeCN	12	28
			Dioxane	81	61
		K_2CO_3	THF	66	52
			MeCN	14	20
			Dioxane	61	44
		K_3PO_4	THF	88	80
			MeCN	12	22
			Dioxane	75	68
		strycine	THF	8	20
			MeCN	11	15
			Dioxane	21	28
	1b	Cs_2CO_3	THF	87	78
			MeCN	10	14
			Dioxane	82	70
		K_2CO_3	THF	45	50
			MeCN	13	22
			Dioxane	78	41
		K_3PO_4	THF	87	80

			MeCN	12	10
			Dioxane	85	70
		strycine	THF	22	20
			MeCN	12	20
			Dioxane	8	24

Pre-catalyst	Allyl Moiety	Base	Solvent	CD *ee*	TLC yield (%)
(R)-C5	1a	Cs_2CO_3	THF	89	80
			MeCN	17	12
			Dioxane	86	64
		K_2CO_3	THF	52	48
			MeCN	12	20
			Dioxane	60	42
		K_3PO_4	THF	91	70
			MeCN	15	28
			Dioxane	90	65
		strycine	THF	25	24
			MeCN	14	16
			Dioxane	20	28
	1b	Cs_2CO_3	THF	90	75
			MeCN	15	32
			Dioxane	89	64
		K_2CO_3	THF	45	49
			MeCN	14	22
			Dioxane	65	38
		K_3PO_4	THF	88	72
			MeCN	18	25
			Dioxane	87	68
		strycine	THF	21	34
			MeCN	14	21
			Dioxane	16	28

Pre-catalyst	Allyl Moiety	Base	Solvent	CD *ee*	TLC yield (%)
(R)-C6	1a	Cs_2CO_3	THF	86	82
			MeCN	18	30
			Dioxane	75	62
		K_2CO_3	THF	44	51
			MeCN	14	12
			Dioxane	60	28
		K_3PO_4	THF	85	66
			MeCN	10	28
			Dioxane	70	57
		strycine	THF	18	11
			MeCN	12	18
			Dioxane	18	21
	1b	Cs_2CO_3	THF	85	77

			MeCN	18	12
			Dioxane	70	58
		K_2CO_3	THF	42	52
			MeCN	10	18
			Dioxane	55	42
		K_3PO_4	THF	84	80
			MeCN	8	16
			Dioxane	48	55
		strycine	THF	15	20
			MeCN	15	21
			Dioxane	18	31

Pre-catalyst	Allyl Moiety	Base	Solvent	CD *ee*	TLC yield (%)
(R)-C7	1a	Cs_2CO_3	THF	82	78
			MeCN	12	22
			Dioxane	75	64
		K_2CO_3	THF	37	61
			MeCN	18	15
			Dioxane	55	22
		K_3PO_4	THF	74	58
			MeCN	15	22
			Dioxane	77	62
		strycine	THF	20	14
			MeCN	16	14
			Dioxane	18	25
	1b	Cs_2CO_3	THF	87	80
			MeCN	9	14
			Dioxane	68	60
		K_2CO_3	THF	44	54
			MeCN	11	16
			Dioxane	59	52
		K_3PO_4	THF	76	72
			MeCN	12	24
			Dioxane	51	58
		strycine	THF	18	21
			MeCN	6	10
			Dioxane	12	27

Pre-catalyst	Allyl Moiety	Base	Solvent	CD *ee*	TLC yield (%)
(R)-C8	1a	Cs_2CO_3	THF	84	80
			MeCN	9	11
			Dioxane	61	58
		K_2CO_3	THF	46	54
			MeCN	17	15
			Dioxane	61	42
		K_3PO_4	THF	84	71

			MeCN	10	8
			Dioxane	77	59
		strycine	THF	15	16
			MeCN	7	10
			Dioxane	10	12
	1b	Cs_2CO_3	THF	84	75
			MeCN	11	16
			Dioxane	66	55
		K_2CO_3	THF	40	43
			MeCN	12	14
			Dioxane	53	52
		K_3PO_4	THF	80	71
			MeCN	14	18
			Dioxane	55	52
		strycine	THF	12	22
			MeCN	10	14
			Dioxane	12	14

Pre-catalyst	Allyl Moiety	Base	Solvent	CD *ee*	TLC yield (%)
(R)-C9	1a	Cs_2CO_3	THF	85	82
			MeCN	11	8
			Dioxane	62	56
		K_2CO_3	THF	40	36
			MeCN	16	18
			Dioxane	61	52
		K_3PO_4	THF	86	78
			MeCN	14	14
			Dioxane	52	55
		strycine	THF	10	12
			MeCN	15	6
			Dioxane	17	15
	1b	Cs_2CO_3	THF	83	78
			MeCN	13	12
			Dioxane	76	55
		K_2CO_3	THF	66	48
			MeCN	14	12
			Dioxane	63	38
		K_3PO_4	THF	84	75
			MeCN	12	12
			Dioxane	55	40
		strycine	THF	18	27
			MeCN	6	9
			Dioxane	11	18
